# Comparison of the Effects of Citicoline and Piracetam on Hypoxic-ischemic Brain Damage in Neonatal Rabbits

**DOI:** 10.22037/ijcn.v15i4.29816

**Published:** 2022-01-01

**Authors:** Sedigheh EBRAHIMI, Soheil ASHKANI ESFAHANI, Alireza EBRAHIMI

**Affiliations:** 1 Department of Medical Ethics, Shiraz University of Medical Sciences, Shiraz, Iran; 2 Department of Orthopaedic Surgery, Massachusetts General Hospital, Harvard Medical School, Boston, Massachusetts, USA; 3 Student Research Committee, Shiraz University of Medical Sciences, Shiraz, Iran

**Keywords:** Citicoline, Rabbits, Hypoxia, Ischemia, Brain damage

## Abstract

**Objectives:**

Perinatal hypoxic-ischemic brain injuries have been a major cause of mortality and neurodevelopmental morbidities in newborns. Citicoline and Piracetam have been used as nootropic agents in a number of studies. In this investigation, we aimed to determine the effects of these agents solely and in combination in hypoxic-ischemic brain damage in rabbit neonates.

**Materials & Methods:**

Hypoxic-ischemic brain damage was induced by the occlusion of both uterine arteries of dams for eight minutes. The subjects were randomly divided into five groups as follows (n=6 per group): control group without hypoxia (C1), control group with hypoxic-ischemic damage (C2), the third group (P) received Piracetam (100 mg/kg), the fourth group (T) administered with Citicoline (250 mg/kg), and the fifth (PT) received both. The preventive effects of the two drugs on hypoxic-ischemic brain damage were microscopically investigated by the rates of damage to the hippocampus.

**Results:**

Neuronal destruction rates in C1, C2, P, T, and PT were 4%, 45%, 37.5%, 12.5% (P=0.01 vs. C2), and 20% (P=0.03 vs. C2), respectively. The total means of hypoxic-ischemic damage, cell edema, neuronal degeneration, and eosinophilic degeneration were lower in the T group compared to C2 (P<0.05).

**Conclusion:**

According to our results and previous findings, Citicoline as a treatment for hypoxic-ischemic brain injuries could be beneficial, and it has priority over neuroprotective agents like Piracetam. Moreover, the combination of Citicoline and Piracetam showed no superior effect in contrast with Citicoline alone. However, experimental studies on larger populations and clinical trials are highly suggested.

## Introduction

Hypoxic-ischemic (HI) brain injury is a condition that occurs when the brain components confront hypoxia, ischemia, and cytotoxicity (1). This disorder typically arises after cardiac arrest, respiratory arrest, incomplete suffocations, poisonous gas exposure, and perinatal asphyxia. Hypoxic-ischemic brain injury frequently produces symptoms such as disturbance of sensorimotor functions, seizure, and impairment of cognitive and emotional tasks (2, 3). The sequelae of the damage depend on the underlying mechanism, the severity, time course, and the duration of oxygen insufficiency and absence of bloodstream (1). 

Perinatal HI brain injuries, such as asphyxia, have been a major cause of mortality in newborn infants and chronic neurodevelopmental deficits in survivors (4). Investigations have reported that about 4 million newborns are affected by moderate to severe asphyxia in the developing countries, 20% of whom suffer from its sequelae, for example, HI encephalopathy (5, 6). However, hypoxic-ischemic encephalopathy (HIE) has a mortality rate of 60%, and its treatment is now limited to intensive supportive care (7-9).

Citicoline is an intermediate complex molecule that involves several vital metabolic pathways (10, 11). Nutritional supplementation and intravenous administration of the agent have shown neuroprotective efficacy in stroke patients and elderly patients with cognitive problems, memory loss, and early stages of Alzheimer’s disease (12-14). Since a few decades ago, Piracetam has been advertised as a nootropic agent in several countries; the drug also has been used for the treatment of cognitive disorders, myoclonus, dyslexia, and vertigo (15, 16). The exact mechanism of Piracetam is yet to be cleared; however, there is growing evidence proving that the agent’s properties rely on the restoration of the cell membrane fluidity (15). Fixed dosage combination of Citocoline and Piracetam (500 mg and 800 mg) is also available in some markets and is used for memory enhancement, neurological and cognitive disorders, Parkinsonism, and Alzheimer’s disease (17). 

Considering the magnitude of the problem of HI brain injuries and the lack of accurate medication for the condition, it is appropriate for health-care professionals to give priority to the issue. Regarding therapeutic interventions and medications, animal studies are important for uncovering the underlying mechanisms of damages and the preventive roles of prescriptions. In this investigation, we aimed to determine the effects of the aforementioned neuroprotective agents solely and in the combination in HI brain-injured rabbits. 

## Materials & Methods

**Animal models**

Rabbits were chosen as the subjects of this experiment mainly due to similarities of their biology with human beings, the high rate of pregnancy, and the high numbers of newborns. Thirty pregnant New Zealand white rabbits, provided from the Animal Laboratory of Shiraz University of Medical Sciences, were randomly selected for the study. They were kept at 25±2°C temperature and in 60% humidity and regular light/dark cycle with adequate food and water for one week before the experiments. 

The models were randomly divided into five groups (n=6). The control group without any hypoxia-ischemia (C1) and the control group with hypoxia-ischemia (C2), which were killed immediately after inducing hypoxia, and their brain sections were used to ensure hypoxia induction and to evaluate the damages. For the third group (P), we prescribed Piracetam (800 mg, Daroupakhsh™, Iran) 100 mg/kg of body weight orally seven days after inducing hypoxia-ischemia. The fourth group (T) received 250 mg/kg of body weight Citicoline (250 mg, Alborzdarou™, Iran) intravenously once just after the hypoxia induction, then they were sacrificed on the seventh day. Finally, the fifth group (PT) received Piracetam (100 mg/kg) orally for a week as well as Citicoline (250 mg/kg) intravenously in a single dose as above. 

**Induction of hypoxic-ischemic brain damage**

Then, the clips were removed for 10 minutes to allow cerebral reperfusion while they were exposed to room air (21% oxygen), and then the fetuses were expulsed. The newborn rabbits were clinically evaluated to ensure their viability (18).

**1. Assessments**

Alive neonates were sacrificed after 10 minutes of exposure to a high dose of pentobarbitone (1 g, Specia™, France). The heads of the neonates were immersion fixed for two weeks in 10% paraformaldehyde in 0.1 M phosphate buffer. Therefore, the brains were systematically cut into 4-mm thick coronal blocks of the hippocampus and were embedded in paraffin and sectioned at 5-micron range with a vibrating microtome. To assess the destruction rate and to determine the presence of cellular hypoxia and degenerating neurons, the sections were stained with hematoxylin and eosin. Changes in Purkinje and pyramidal cells and necrosis, and edema in the damaged areas were determined under a light microscope (×400) by a pathologist who was blinded to the experimental groups. Analysis was performed by a light microscope, which showed Purkinje and pyramidal cells’ deformities as well as necrosis and edema in the studied regions. The preventive effects of the two drugs on hypoxia-ischemia were investigated by the rates of damage to hippocampal neurons.

**2. Ethical considerations**

All the experiments were performed according to the Guide for the Care and Use of Laboratory Animals (NIH publication No. 86-23, 1985 edition) approved by the Ethics Committee of Shiraz University of Medical Sciences.

**3. Statistical analysis**

Histopathological scores were designed as median range and mean on graphs by GraphPad Prism software; also, they were analyzed using a non-parametric Mann-Whitney U test by SPSS software (version 21.0, Chicago, IL, USA). A *P-*value of less than 0.05 was considered statistically significant. 

## Results

Neuronal destruction in the C1 group was about 4%, and the C2 group showed about 45% damage. In the T group, the rate of destruction was about 12.5%, which was significantly lower than that of C2. The destruction rate of the P group was near 37.5%, and the rate of destruction in the PT group was about 20-25% (Table 1). The results indicated that administrating intravenous Citicoline alone or in combination with Piracetam can decrease cell death in comparison to the untreated hypoxic-ischemic brain-injured control group (P ≤ 0.05). Citocoline could also noticeably decline brain edema (P ≤ 0.001) besides the total average of hypoxic-ischemic brain damage (P ≤ 0.05). Although the PT group showed a considerably lower damage of neuronal degeneration, the total average of hypoxic-ischemic damage in the P group and PT group was 36.3±4.42% and 27.9±10.94, respectively, which were not significantly different from that of the C2 group. Moreover, there was no significant reduction in the brain edema of the P and PT groups, and the degrees of brain edema were approximately near that of the control group (P > 0.05).

**Table1:** Intensity of the hypoxic-ischemic damage in the hippocampus of the rabbits of control group without any hypoxia (C1), control group with hypoxia (C2), experimental group with hypoxia treated with Piracetam (P), experimental group with hypoxia treated with Citicoline (T) and the experimental group with hypoxia treated with Piracetam and Citicoline (PT)

Total average of hypoxic ischemic damage %	Numbers of rabbits	Average of cell degeneration %	Average of cell edema %	Average of eosinophilic degeneration %	Groups
1±3	5	4	3	2	Group C1
38.2±11.55	6	45	45	25	Group C2
22.9±13.02	6	12.5	37.5	18.7	Group T
36.3±4.42	6	37.5	40	18.7	Group P
27.9±10.94	6	25	40	18.7	Group PT

* P≤0.05 vs. C2 group

† P≤0.05 vs. C1 group

## Discussion

Hypoxic-ischemic brain injury in general, and specifically, neonatal encephalopathy due to perinatal HI conditions are associates with high mortality, morbidity, and chronic lifelong disabilities (18). Currently, the treatment of HIE in newborns is limited to intensive supportive care, such as the reduction of whole-body temperature and brain temperature (9). However, reports have suggested that cell therapy and some drugs such as Alluporinol and Astragalus could be used in the case of perinatal HI brain damage (19-21). 

Our study showed that rabbit neonates who suffered from HI brain damage and were treated with Citocoline had a significantly lower degeneration and edema of neuronal cells besides lower total average HI destruction of the brain in comparison with the ones that did not receive the medication. The results were consistent with the findings of a prior study performed by Rao et al., which showed that the drug had protective efficacy against blood-brain barrier (BBB) dysfunction in the forebrain of gerbils. Başkaya et. al reported a significant reduction in brain edema and BBB breakdown after using the agent in rats affected by traumatic brain injury (22). Similar perinatal asphyxia models of rats indicated that the drug had a dose-dependent neuroprotective effect (23). Another study also supported the hypothesis of prescribing Citocoline for reducing the brain lesion growth produced by ischemic strokes by using diffusion-weighted magnetic resonance imaging (24). Investigations have reported that newborn nerve action (NBNA) scores in newborns suffering from HIE and gross motor function classification system (GMFCS) levels were improved after using the drug (25, 26).

Piracetam and Piracetam-like drugs have a vast range of applications in neurological problems, for example, cognition/memory deficits, myoclonus epilepsy, ataxia, aphasia, chronic fatigue syndrome, and autism. A meta-analysis on animal models of stroke and cerebral ischemia also mentioned that the drug could have beneficial effects on the improvement of consequences (27). As our study demonstrated, Piracetam also had a neuroprotective effect by reducing average neuronal cell degeneration in rabbits’ hippocampus. However, no significant reduction was observed in BBB failure and cell edema of the specimen. A previous study on rats with chronic cerebral hypo-perfusion demonstrated that the agent could improve memory deficits and neuronal damages (28). Kessler et. al reported that Piracetam facilitated the recovery of verbal skills in post-stroke patients; they also showed a significant increase in task-related flow activation in the left hemisphere of the brain after using the medication (29). 

A combined tablet of Citocoline and Piracetam is recently introduced to the market for several neurologic problems. However, combined administration of the drugs decreased neuronal cell degeneration by 20%; there was no major decline in cell edema in the hippocampus of HI brain-injured rabbits. Consistently, a previous study also reported that the combined administration of these medicines only elevated serotonin, among different factors, in the hippocampus of rats (30). 

The application of the drugs was started by the induction of HI brain damage; tough, the narrow lifespan of rabbits after HI brain damage restricted the follow-up of the study models to more than one week. The strength of data resulting from this animal model and their association with humans is also debatable. Large investigations and confirmatory results are needed to elucidate the exact effects of these drugs in HI brain damage conditions.

Our study demonstrated the neuroprotective effects of Citocoline and Piracetam in HI brain damage in neonatal rabbits. The animal model was designed to mimic perinatal asphyxia and HI encephalopathy in newborns. We concluded that regarding the low side effects of these drugs, Citocoline and Piracetam could be used in the aforementioned conditions. Citocoline could have priority in this regard, as our investigation showed the superior efficacy of this drug. However, larger studies and clinical trials are needed to approve the prescription of these drugs under HI brain injury circumstances.

## Author’s contribution 

Study design and hypothesis: Sedigheh Ebrahimi

Experiments and data gathering: Soheil Ashkani Esfahani and Sedigheh Ebrahimi

Data and lab analyses, organization, writing and editing the draft of the paper: Soheil Ashkani-Esfahani, Alireza Ebrahimi

All the authors have participated in final editing of the drafts and have reviewed the final version of the paper.

## Conflicts of Interest

The authors declare that there is no conflict of interest regarding the publication of this paper.
